# Notch signaling inhibitor DAPT provides protection against acute craniocerebral injury

**DOI:** 10.1371/journal.pone.0193037

**Published:** 2018-02-15

**Authors:** Hong-Mei Zhang, Pei Liu, Cheng Jiang, Xiao-Qing Jin, Rui-Ning Liu, Shun-Qing Li, Yan Zhao

**Affiliations:** 1 Emergency Center, Zhongnan Hospital of Wuhan University, Wuhan, Hubei, China; 2 Department of Intensive Care Unit, Taihe Hospital, Hubei University of Medicine, Hubei, China; Fraunhofer Research Institution of Marine Biotechnology, GERMANY

## Abstract

Notch signaling pathway is involved in many physiological and pathological processes. The γ-secretase inhibitor DAPT inhibits Notch signaling pathway and promotes nerve regeneration after cerebral ischemia. However, neuroprotective effects of DAPT against acute craniocerebral injury remain unclear. In this study, we established rat model of acute craniocerebral injury, and found that with the increase of damage grade, the expression of Notch and downstream protein Hes1 and Hes5 expression gradually increased. After the administration of DAPT, the expression of Notch, Hes1 and Hes5 was inhibited, apoptosis and oxidative stress decreased, neurological function and cognitive function improved. These results suggest that Notch signaling can be used as an indicator to assess the severity of post-traumatic brain injury. Notch inhibitor DAPT can reduce oxidative stress and apoptosis after acute craniocerebral injury, and is a potential drug for the treatment of acute craniocerebral injury.

## Introduction

The high mortality rate and neurological deficits caused by brain injury bring huge economic burden to the family and society [1]. At present the treatments of traumatic brain injury include preventing cerebral edema, reducing intracranial pressure and sub-hibernation and other conservative treatment methods, but the treatment outcomes are not satisfactory [2]. The protection of neurons and restoration of their function are essential to the treatment of brain injury. Therefore, the development of new neuroprotective drugs is important to the treatment of brain injury.

Notch signaling pathway is involved in many key physiology and pathological processes by regulating intercellular contact-dependent communication, cell differentiation, proliferation and apoptosis, and determining cell fate [3]. The extracellular domain of Notch receptor binds to Notch ligand on the adjacent cell surface and triggers signal transduction. When the Notch receptor interacts with the ligand, the γ-secretase complex catalyzes protein cleavage in the transmembrane region of Notch receptor, releases Notch intracellular domain (NICD) from the inside of cell membrane. NICD directly enters the nucleus after the release and interacts with transcription factor RBP-J to induce the expression of downstream target genes such as Hes [4]. Therefore, γ-secretase inhibitor can specifically inhibit the activation of Notch signaling [5]. Several studies have shown that γ-secretase complex and Notch1 are involved in the pathogenesis of nervous diseases such as Alzheimer's disease and ischemic stroke. DAPT is an inhibitor of Notch signaling that promotes neurological regeneration after cerebral ischemia and exhibits neuroprotective effect [6]. However, the role of Notch signaling in traumatic brain injury remains unclear.

The pathogenesis of traumatic brain injury (TBI) is complex and involves early mechanical damage, oxidative stress, inflammatory response, neuronal cell apoptosis and secondary neurodegeneration [7–9]. Since Notch signaling could regulate oxidative stress and apoptosis, we speculated that acute craniocerebral trauma could activate Notch signaling to imitate consequent damages. Rat model of TBI could mimic the situation in human. Therefore, in this study we established rat model of TBI to investigate whether Notch signaling is involved in the development of acute craniocerebral trauma, and whether Notch inhibitor DAPT could protect against acute craniocerebral trauma.

## Materials and methods

### Animals

Specific-pathogen-free (SPF) grade SD male rats (14–15 weeks old, 250–300 g weight) were purchased from Animal Center of Wuhan University. Animal experiment was approved by Animal Experiment Center and ethics committee of Zhongnan Hospital of Wuhan University. DAPT solution (1 μg/μl) was prepared by dissolving DAPT powder (MCE, USA) in 0.01 M phosphate buffered saline (PBS) containing 5% dimethyl sulfoxide [10]. The solution was filtered and stereotactically injected into the right cerebral ventricle using the following coordinates: -0.8 mm anteroposterior, ±1.6 mm mediolateral, and -4.0 mm dorsoventral from the bregma [11]. Control rats received the injection of PBS instead of DAPT in the same way.

### Animal model

**Fig 1:**
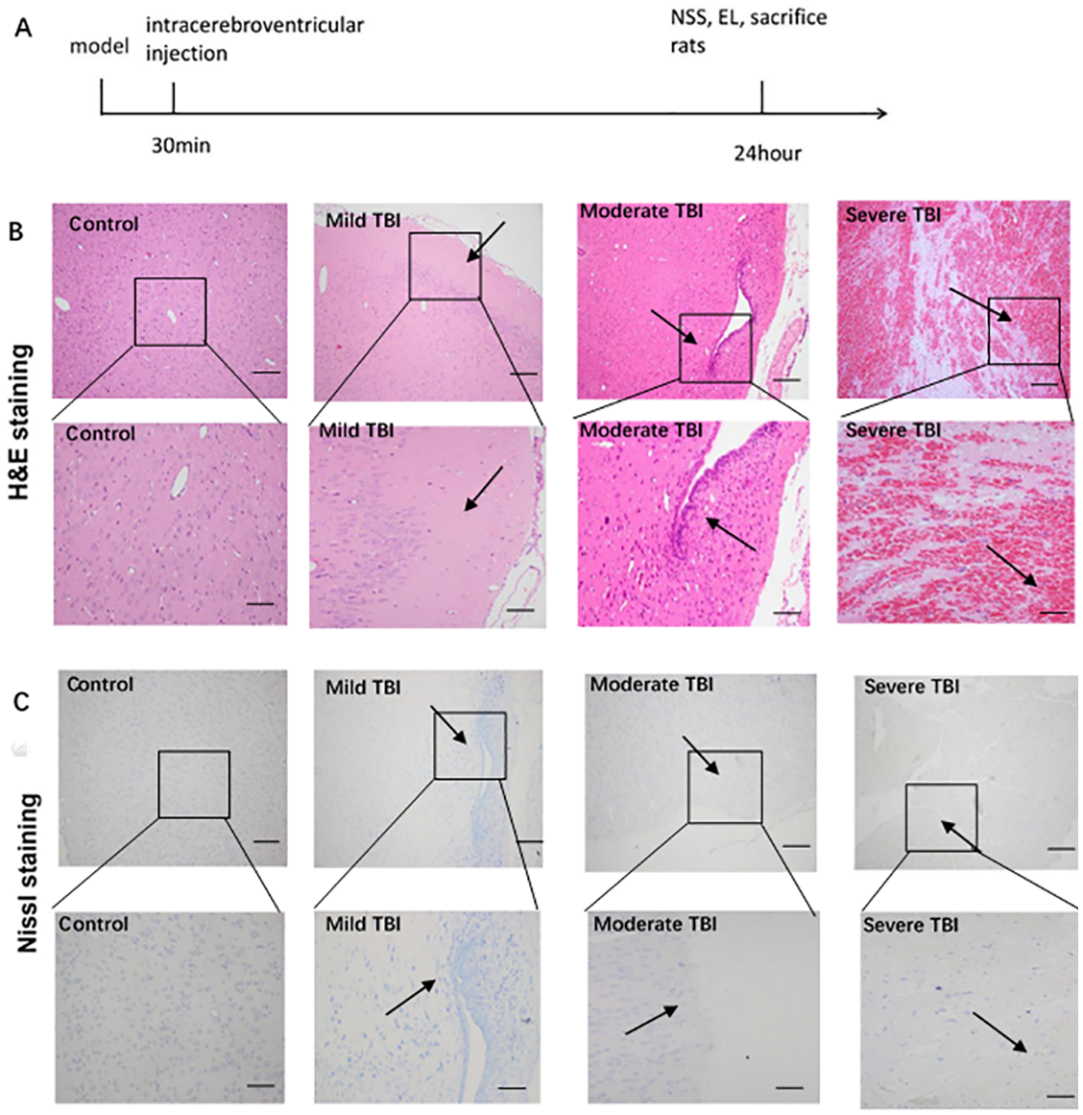
A. Scheme of the timeline for the treatment of the rats in this study. B. H&E staining. No obvious damage was observed in Control group, while the damaged regions in other groups were indicated by the arrows. C. Nissl staining. Nissl staining was normal in Control group, while the regions with loss of Nissl body in other groups were indicated by the arrows. Shown were representative images from three rats in each group. TBI = traumatic brain injury. Scale bar: 100 μm for whole pictures. Scale bar: 20 μm for inserted pictures.

Animal models with different degree of traumatic brain injury (TBI) were prepared as described previously [12]. Briefly, the rats received intraperitoneal injection of 1% pentobarbital at the dose of 30 mg/kg. After the success of the anesthesia, rats were fixed on the brain stereotaxic device to cut the top of the skull skin to determine the bregma. Then a diameter of 5 mm bone window was drilled, and dura mater was exposed. A 20 g hit hammer fell at 10 cm vertically along the outer tube leading to mild TBI, while a 40 g hammer fell at 15 cm or 25 cm vertically along the outer tube leading to moderate or severe TBI, respectively. Control group received no brain damage. 30 min after injury, DAPT or PBS was injected into lateral ventricle, followed by the closing of bone window. Experimental grouping was as follows (n = 10): control group, mild TBI, moderate TBI, severe TBI, severe TBI+DAPT, severe TBI+PBS. 24 h later, the rats were analyzed for function performance and then sacrificed (Fig 1A).

### Neurological function

The neurological function of the rats was evaluated using Neurological Severity Scores (NSS) as described previously [13]. One point is awarded for the inability to perform the tasks or for the lack of a tested reflex; 13 to 18 indicates severe injury; 7 to 12, moderate injury; 1 to 6, mild injury [13].

### Water maze test

The learning and memory abilities of rats were evaluated by recording escape latency. Water maze test was performed 24 h after TBI. The rats in each group were trained in water maze four days and four times a day. The time of searching for platform of each rat was recorded as escape latency and the mean was calculated.

### Histological analysis

A small incision was cut in right auricle of anesthetized rat, after left ventricular catheterization, the rats received perfusion with 150 ml paraformaldehyde. The brain was dissected, paraffin embedded, and cut into 5 μm sections. The sections were stained by hematoxylin-eosin (HE) and Nissl staining following routine procedures and observed under microscope.

### TUNEL

Apoptosis of neurons was detected by TUNEL staining, which was performed using Apoptosis Detection Kit (Roche Applied Science) following the manufacturer’s instructions. The number of TUNEL-positive cells was counted under fluorescence microscope.

### Western blot analysis

Total protein was isolated from brain tissues and quantitated by BSA method. 30 μg protein was separated on 10% SDS-PAGE and transferred to PVDF membranes (Millipore, Billerica, MA, USA). Next, the membranes were incubated at 4°C overnight with following primary antibody: GAPDH (Cat.No. 10494-1-AP, 1: 1,000 dilution), Notch1 (Cat.No. 10062-2-AP, 1:1,000 dilution), Hes5 (Cat.No. 22666-1-AP, 1:500 dilution), Bcl2 (Cat.No. 12789-1-AP, 1:1,000 dilution), Bax (Cat.No. 23931-1-AP, 1:1,000 dilution), caspase 3 (Cat.No. 19677-1-AP, 1:600 dilution) and caspase 9 (Cat.No. 10380-1-AP, 1:1,000 dilution) (all from Proteintech Inc.), and Hes1 (Cat.No. 2922–1, 1:800 dilution) (Epitomics, CA, USA). The membranes were washed with TBST three times, then incubated with HRP labeled goat anti-rabbit secondary antibody (Cat.No. sc-2004, Santa Cruz Biotech., Santa Cruz, CA, USA) for 1 h at room temperature. The membranes were developed using ECL kit (Pierce, Rockford, IL, USA) and exposed to X-ray film.

### Real-time PCR

Total RNA was extracted from the cortex using TRIzol reagent (Invitrogen, USA) following the manufacturer’s protocol. cDNA was produced by reverse transcription using RT kit (Promega, Madsion, WI, USA) following the manufacturer’s protocol. PCR was performed using Taq Master Mix (Promega, Madison, WI, USA). The primers used were as follows: Rat GAPDH 5‘-ACAGCAACAGGGTGGTGGAC -3’ (forward) and 5‘-TTTGAGGGTGCAGCGAACTT -3’ (reverse); Rat Notch 5‘- TGGCTCCATCGTCTACCT -3’ (forward) and 5‘- TCCACCGTCTCACTCTTTAC-3’ (reverse); Rat Hes1 5‘- TTGGCGGCTTCCAAGTGGTG-3’ (forward) and 5‘- GGTCCCGCTGTTGCTGGTGTA-3’ (reverse); Rat Hes5 5‘- AGCCAGCGACACGCAGATGA-3’ (forward) and 5‘- CCAGAGGCCGCAGGCAGATT-3’ (reverse); Rat Bax 5‘- CAGGCGAATTGGCGATGAAC-3’ (forward) and 5‘- CCCAGTTGAAGTTGCCGTCT-3’ (reverse); Rat BCL2 5‘- GGGGAGCGTCAACAGGGAGA-3’ (forward) and 5‘- AGACAGCCAGGAGAAATCAA-3’ (reverse); Rat Caspase 3 5‘-GGACCTGTGGACCTGAAAAA-3’(forward) and 5‘-GCATGCCATATCATCGTCAG-3’(reverse); Rat Caspase 9 5‘-CACTGCCTCATCATCAACAAC-3’ (forward) and 5‘-TGTGCCATAGACAGCACCC-3’ (reverse). Amplification conditions were as follows: 95°C 10 min for one cycle; then 95°C 30 sec and 60°C 30 se for 40 cycles.

Relative mRNA levels were calculated using 2^-ΔΔCt^ method.

### MDA and total SOD detection

The contents of total SOD and MDA were measured by xanthine oxidase method and thiobarbituric acid method, respectively. The brain tissues were homogenized on ice and then centrifuged at 3,000 rpm for 10 min. The supernatants were taken, and total SOD and MDA contents were detected using SOD assay kit (Cat.No. A001-1, Nanjing Jiancheng Bioengineering Institute, Nanjing, China) and MDA assay kit (Cat.No. A003-1, Nanjing Jiancheng Bioengineering Institute, Nanjing, China) in accordance with the instructions.

### Statistical analysis

All data were expressed as mean ± SD and analyzed by SPSS18.0 software (SPSS Inc., Chicago, IL, USA). Statistical differences between groups were determined using one-way ANOVA followed by student t-test. A two tailed *p* value less than 0.05 was considered statistically significant.

## Results

### Pathological manifestations of brain trauma in different groups

The damage of the right side of the parietal lobe was observed in the injured groups. With the increase of the impact force, the area and severity of the injury increased, the swelling degree of severe TBI increased. In contrast, control group had no signs of injury and brain tissue swelling. HE staining showed no pathological changes in control group. In TBI groups we observed disordered and sparse structure of damage area, and red blood cells dispersed in the damaged tissue. In mild TBI the damage was mainly confined to the cortex with a small amount of bleeding. In moderate TBI the damage aggravated, deep to the cortex and a small amount of hippocampus. In severe TBI, injury area and extent significantly aggravated, involving the hippocampus and a small amount of contralateral brain tissue, and edema significantly aggravated (Fig 1B). Nissl staining showed that Nissl body was normal in control group, but was not observed in TBI lesion regions, and the extent of loss of Nissl body correlated with the increase of injury severity (Fig 1C).

### Apoptosis in injury area in different groups

**Fig 2:**
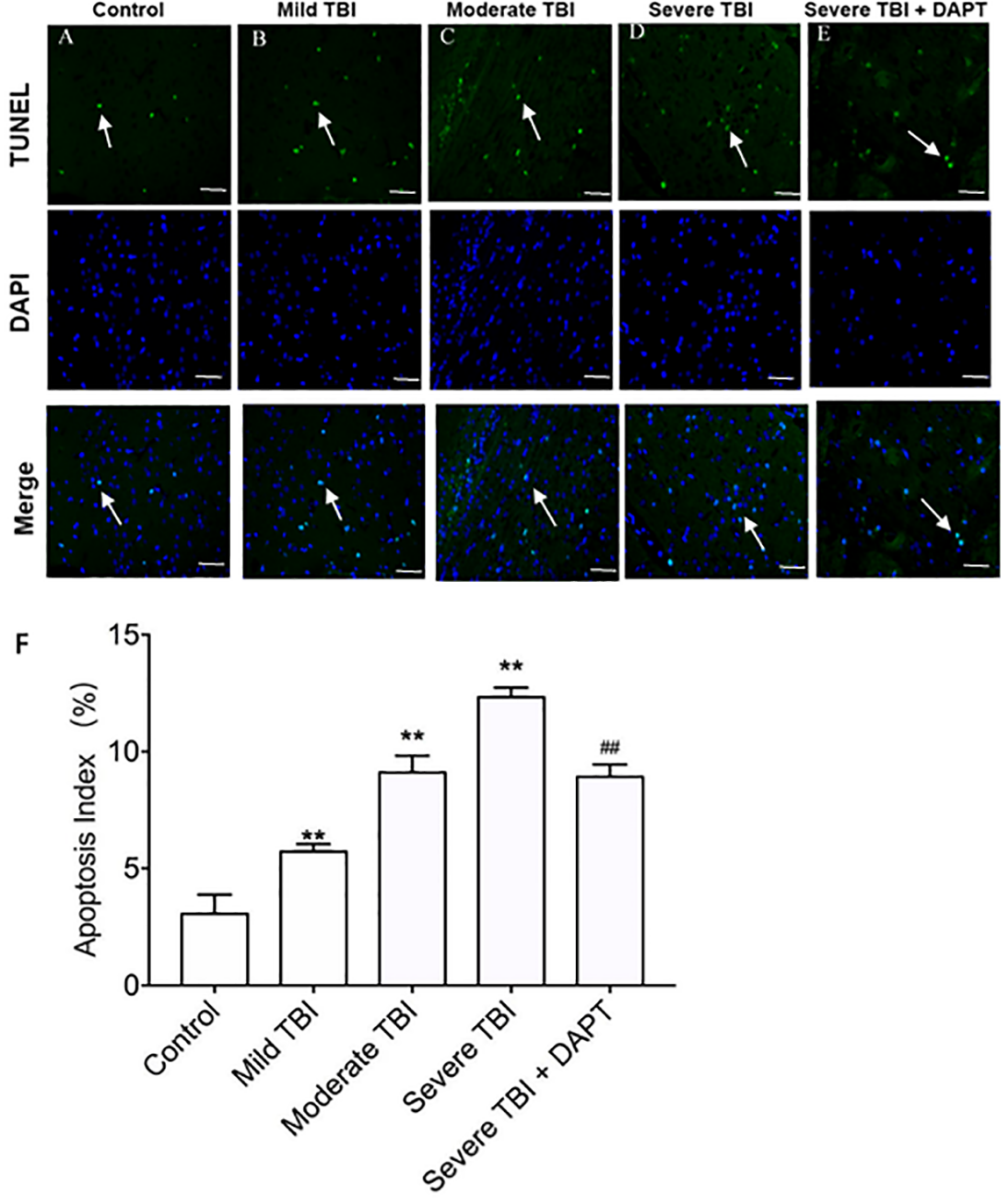
TUNEL staining of cortical area. A. Control group. B. Mild TBI group. C. Moderate TBI group. D. Severe TBI group. E. Severe TBI + DAPT group. Scale bar: 100 μm. F. Apoptosis index. Apoptotic cells were stained as green (indicated by the arrows), while the nucleus were stained as blue. With the increase of TBI severity, more apoptotic cells were observed, but DAPT treatment reduced the number of apoptotic cells in Severe TBI group. Shown were representative images from three rats in each group. All data were expressed as mean ± SD (n = 3). **p < 0.01 vs. Control group, ^##^*p* < 0.01 vs. severe group. TBI = traumatic brain injury.

There were significant differences in apoptotic cells in injury area among different groups (Fig 2A–2E, F = 144.768, n = 3 animals per group, P<0.01). The number of apoptotic cells significantly increased in severe TBI group, but decreased after DAPT treatment (Fig 2F, T = 2.414, n = 3 animals per group, P<0.01).

### Neurological function in different groups

**Fig 3:**
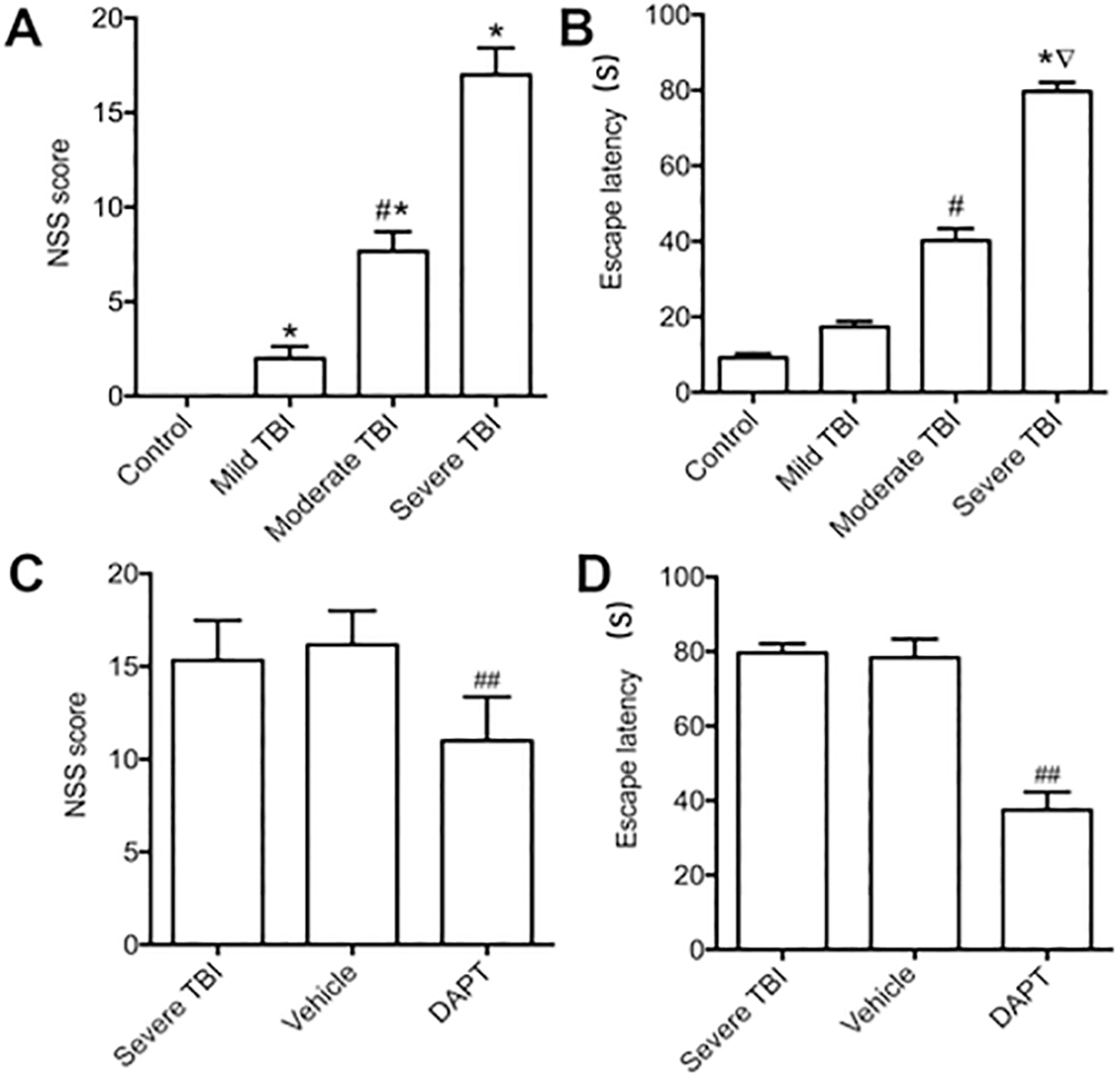
Functional analysis of the rats in all groups. NSS score (A) and Escape latency (B) in all groups. NSS score and escape latency were increased with the severity of TBI. NSS score (C) and Escape latency (D) of Severe TBI rats treated with vehicle or DAPT. Treatments with DAPT significantly decreased the NSS score and escape latency. All data were expressed as mean ± SD (n = 3). *p < 0.01 vs. Control group; ^∇^*p* < 0.01 vs. mild group; ^#^*p* < 0.05, ^##^*p* < 0.01 vs. other groups. Escape latency was measured and expressed as second. NSS = Neurological Severity Scores. TBI = traumatic brain injury.

There were significant differences in NSS scores among different groups of brain injury. With the increase of injury severity, NSS score became higher. Severe injury group had the highest NSS score, suggesting the most serious neurological deficit (Fig 3A, F = 230.851, n = 10 animals per group, P<0.01). After DAPT treatment, NSS score significantly improved in severe injury group (Fig 3C, T = 3.714, n = 10 animals per group, P<0.01), suggesting that DAPT could improve neurological deficit after traumatic brain injury. Furthermore, Water maze test showed that escape latency in TBI group was longer than in control group, and it significantly extended with the increase of injury severity (Fig 3B, F = 9.135, n = 10 animals per group, P<0.01). The escape latency was shortened after the treatment with DAPT (Fig 3D, T = 5.881, n = 10 animals per group, P<0.01), suggesting that DAPT could improve cognitive and memory abilities after TBI.

### DAPT inhibits Notch signaling and regulates apoptosis associated proteins in TBI group

**Fig 4:**
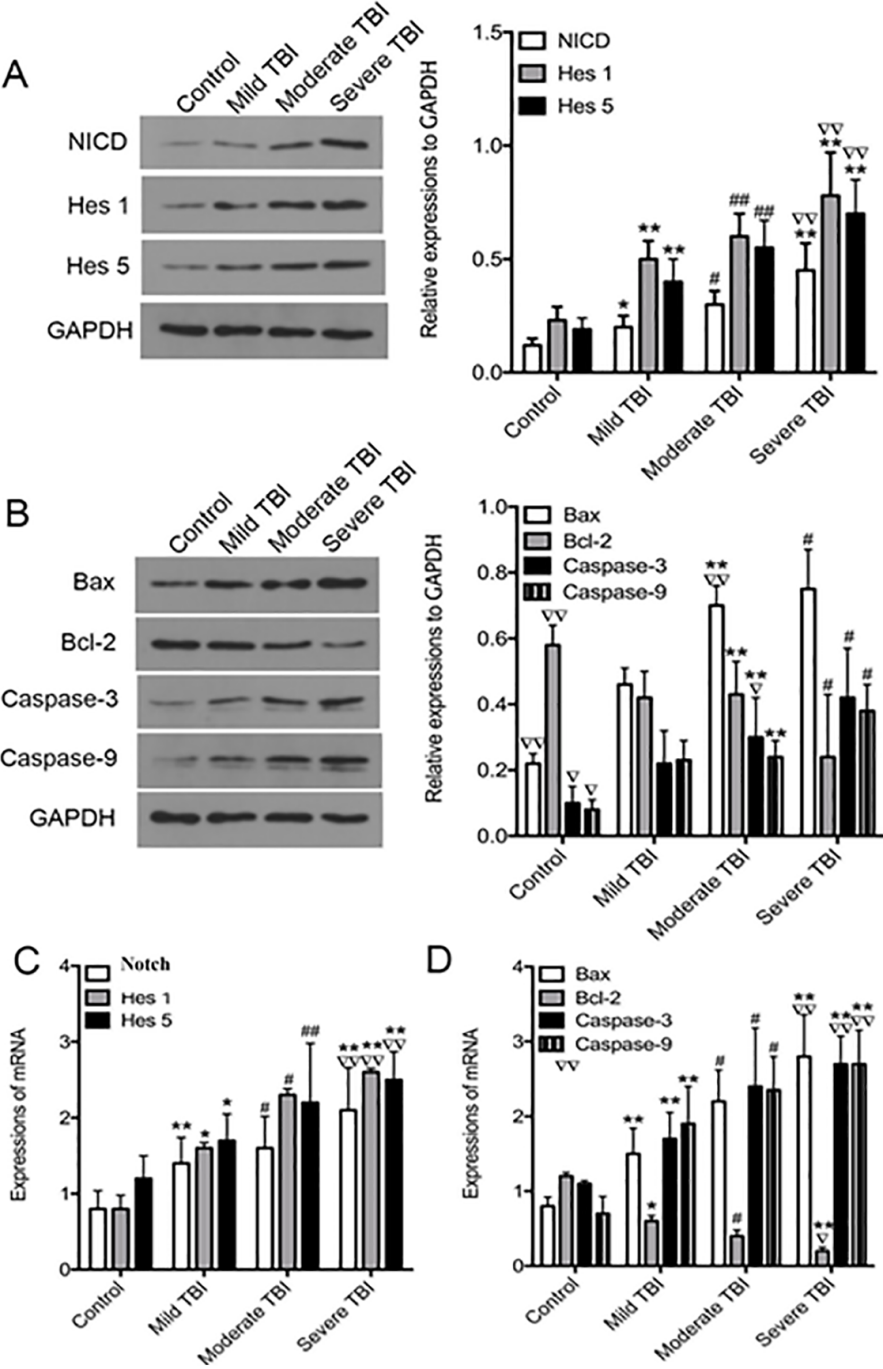
Protein and mRNA expression of Notch, Hes 1, Hes 5 and apoptosis related proteins. A. Western blot analysis of protein levels of NICD, Hes1 and Hes5. B. Western blot analysis of protein levels of apoptosis related proteins. C. PCR analysis of mRNA levels of Notch, Hes1 and Hes5. D. PCR analysis of mRNA levels of apoptosis related proteins. All data were expressed as mean ± SD (n = 3). *p < 0.05, **p < 0.01 vs. Control group; ^∇^*p* < 0.05, ^∇∇^*p* < 0.01 vs. mild group; ^#^*p* < 0.05, ^##^*p* < 0.01 vs. other three groups. NICD = Notch intracellular domain.

Western blot analysis showed that with the increase of injury severity, protein levels of Notch and downstream proteins Hes1 and Hes5 increased significantly. The expression of Notch, Hes1 and Hes5 proteins in severe injury group were the highest, and the differences were statistically significant (Fig 4A, F = 31.110 for NICD, F = 26.000 for Hes1, F = 22.284 for Hes5, n = 3 animals per group, P<0.01). Similarly, real-time PCR showed that with the increase of injury severity, mRNA levels of Notch, Hes1 and Hes5 increased significantly. The expression of Notch, Hes1 and Hes5 mRNAs in severe injury group were the highest, and the differences were statistically significant (Fig 4C, F = 28.039 for Notch, F = 26.573 for Hes1, F = 28.791 for Hes5, n = 3 animals per group, P<0.01).

The expression of apoptosis inhibitory factor protein Bcl-2 in each group gradually decreased with the increase of injury severity, Bcl-2 protein and mRNA levels were the lowest in severe injury group. In contrast, the expression of pro-apoptotic factor Bax, caspase-3 and caspase-9 increased with the increase of injury severity. The protein levels of Bax, caspase-3 and caspase-9 were the highest in severe brain injury group, and the differences were statistically significant (Fig 4B, F = 23.805 for Bax, F = 5.859 for Bcl-2, F = 23.546 for Caspase-3, F = 36.817 for Caspase-9, n = 3 animals per group, P<0.01). Similarly, the mRNA levels of Bax, caspase-3 and caspase-9 were the highest in severe brain injury group, and the differences were statistically significant (Fig 4D, F = 25.790 for Bax, F = 20.402 for Bcl-2, F = 19.885 for Caspase-3, F = 21.397 for Caspase-9, n = 3 animals per group, P<0.01).

**Fig 5:**
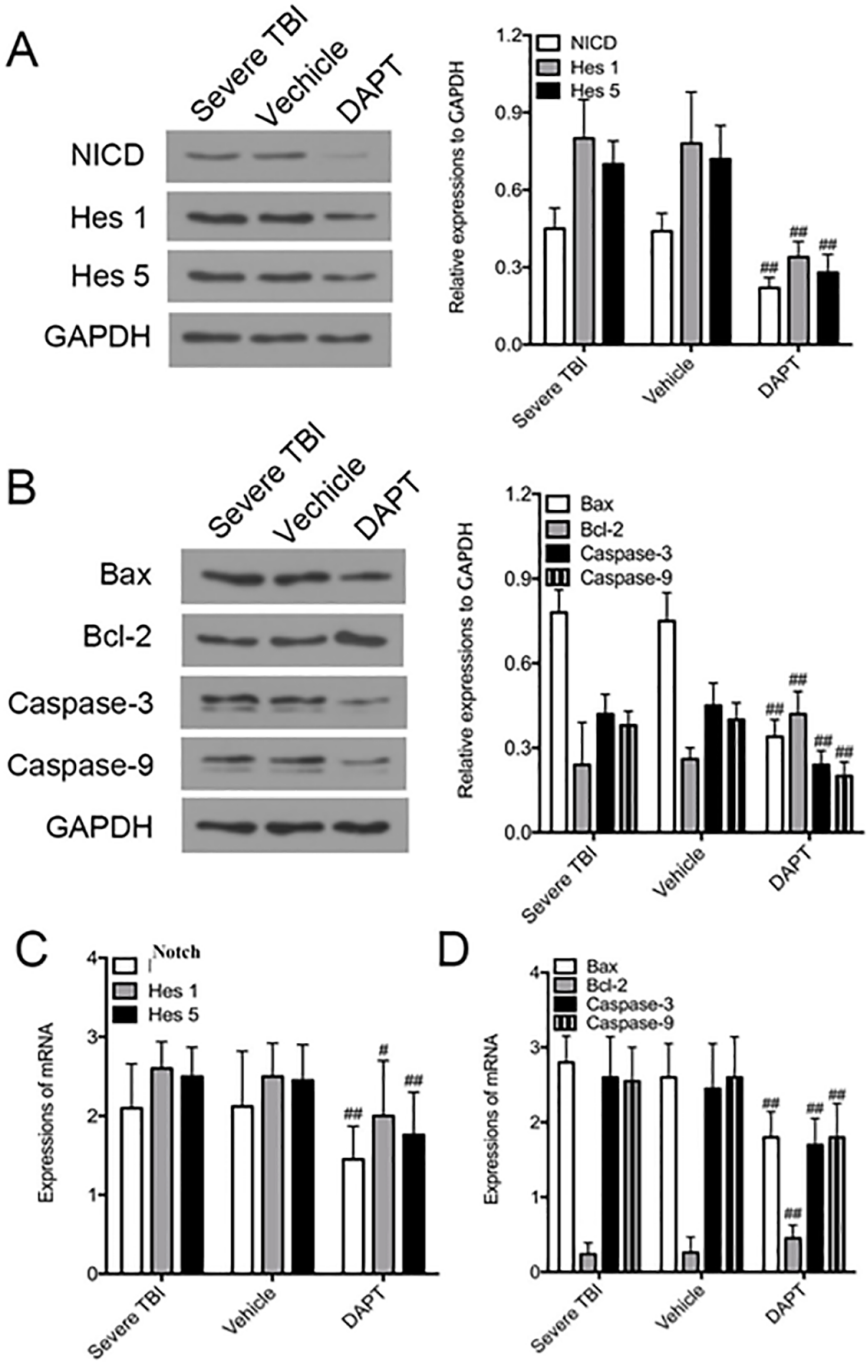
Protein and mRNA expression of NICD, Hes1, Hes5 and apoptosis related proteins in severe TBI rats treated with DAPT. A. Western blot analysis of protein levels of NICD, Hes1 and Hes5. B. Western blot analysis of protein levels of apoptosis related proteins. C. PCR analysis of mRNA levels of Notch, Hes1 and Hes5. D. PCR analysis of mRNA levels of apoptosis related proteins. All data were expressed as mean ± SD (n = 3). *p < 0.05, **p < 0.01 vs. Control group; ^∇^*p* < 0.05, ^∇∇^*p* < 0.01 vs. mild group; ^#^*p* < 0.05, ^##^*p* < 0.01 vs. other three groups. NICD = Notch intracellular domain. TBI = traumatic brain injury.

After DAPT treatment, the protein levels of Notch, Hes1 and Hes5 were downregulated in severe injury group (Fig 5A, T = 3.690 for NICD, T = 3.387 for Hes1, T = 3.969 for Hes5, n = 3 animals per group, P<0.01). Similarly, mRNA levels of Notch, Hes1 and Hes5 were downregulated in severe injury group (Fig 5C, T = 2.637 for Notch, T = 2.516 for Hes1, P<0.05; T = 3.725 for Hes5, n = 3 animals per group, P<0.01). After DAPT treatment, the protein levels of Bcl-2 were upregulated while those of Bax, caspase-3 and caspase-9 were downregulated (Fig 5B, T = 3.832 for Bax, T = 3.674 for Caspase-9, n = 3 animals per group, P<0.01; T = 3.049 for Bcl-2, T = 2.861 for Caspase-3, n = 3 animals per group, P<0.05). Similarly, mRNA levels of Bcl-2 were upregulated while those of Bax, caspase-3 and caspase-9 were downregulated (Fig 5D, T = 3.168 for Bax, T = 3.589 for Caspase-3, n = 3 animals per group, P<0.01; T = 2.217 for Bcl-2, T = 3.133 for Caspase-9, n = 3 animals per group, P<0.05). Taken together, these data suggest that DAPT inhibits Notch signaling and antagonizes apoptosis in the brain subjected to TBI.

### DAPT relieves oxidative stress in TBI group

**Fig 6:**
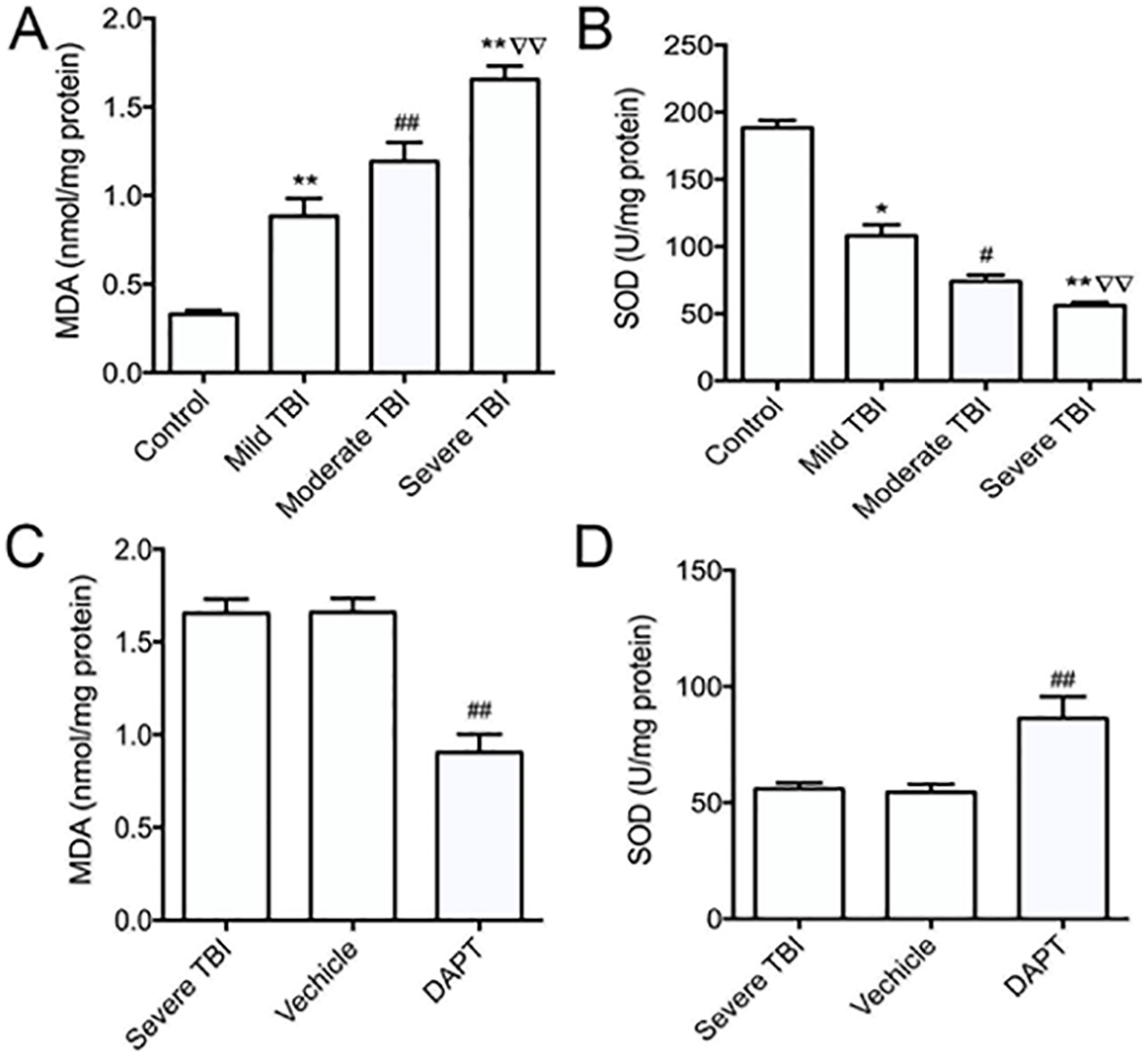
DAPT treatment reduced oxidative stress in TBI rats. MDA expressions (A) and SOD levels (B) in the cortex of all groups. MDA expressions (C) and SOD levels (D) in the cortex of severe TBI rats treated with or without DAPT. All data were expressed as mean ± SD (n = 3). *p < 0.05, **p < 0.01 vs. Control group; ^∇∇^*p* < 0.01 vs. mild group; ^#^*p* < 0.05, ^##^*p* < 0.01 vs. other three groups. MDA = Malondialdehyde. SOD = superoxide dismutase.

With the increase of injury severity, total MDA level in the cortical tissue around the injury increased gradually (Fig 6A, F = 24.530, n = 3 animals per group, P<0.01), while total SOD level decreased gradually (Fig 6B, F = 29.043, n = 3 animals per group, P<0.01). After DAPT treatment, total MDA level decreased significantly (Fig 6C, T = 6.341, n = 3 animals per group, P<0.01), while total SOD level increased significantly in severe injury group (Fig 6D, n = 3 animals per group, T = 3.634, P<0.01).

## Discussion

The incidence of craniocerebral injury is increasing, and it ranks the first in the morbidity and mortality after injury [14]. Current drugs, physical therapy and other treatments could not achieve satisfactory efficacy to treat craniocerebral injury. Therefore, how to improve the recovery of neurological dysfunction after traumatic brain injury will be important.

Brain tissue lacks adequate antioxidant protection against free radical damage. When free radicals overcome cellular antioxidant defense, oxidative stress will occur. The brain is rich in polyunsaturated fatty acids and low in antioxidant enzymes, thus becomes vulnerable to free radical damage. Free radicals are widely involved in brain edema, cerebral hemorrhage, brain trauma and other neurological diseases [15]. In this study, we found that MDA content increased in rat brain subjected to different extent of brain injury, and was the highest in severe traumatic brain injury group. With the increase of injury severity, the content of antioxidant SOD decreased gradually, and SOD content was the lowest in severe traumatic brain injury group. These data suggest that oxidative stress is involved in the development of brain damage after TBI.

The death of neuronal cells after TBI includes primary nerve cell death and secondary nerve cell death. Primary nerve cell death is a direct cell death caused by physical injury, which is irreversible, while 10–50% of secondary neuronal cell death is neuronal apoptosis [16–18]. In this study, TUNEL assay showed that with the increase of injury severity, the number of apoptotic cells increased. The mRNA and protein expression of pro-apoptotic factor Bax, Caspase-3 and Caspase-9 increased gradually, while the expression of Bcl-2 decreased gradually, indicating that apoptosis is involved in the pathogenesis of TBI. Therefore, intervening with gene expression involved in the apoptosis by different means is expected to provide a new strategy to treat brain injury and recovery neuron function.

Notch signaling plays an important role in cell fate determination, differentiation, proliferation, apoptosis, adhesion and epithelial-mesenchymal transformation [19,20]. In this study, we found that the expression of Notch, Hes1 and Hss5 increased after acute craniocerebral trauma, and was positively correlated with injury severity, which indicated that Notch signaling could be used as a reliable indicator to assess acute brain injury. Several studies have shown that Notch inhibitor could improve neurological damage in rats with cerebral ischemia [21]. However, other studies have shown that the activation of Notch signaling can protect the nervous system [22]. Whether Notch signaling activation plays a protective or damage role in acute traumatic brain injury remains unclear. In our preliminary experiments we found that Notch inhibitor DAPT could improve mild and moderate TBI, but DAPT showed significant improvement on severe TBI. Therefore, in this study we focused on the effects of DAPT on severe TBI. We found that in the acute stage of traumatic brain injury, DAPT improved neurological function score and cognitive function of the rats, suggesting that Notch signaling may aggravate acute traumatic brain injury. In addition, DAPT treatment decreased the number of apoptotic cells, decreased the expression of pro-apoptotic factor Bax, Caspase-3 and Caspase-9, and increased the expression of apoptosis inhibitory factor Bcl-2, which indicated that Notch signaling inhibitor could play a neuroprotective role by inhibiting neuron apoptosis. These data are consistent with previous studies that Notch signaling could promote neuronal death during cerebral ischemic by enhancing the apoptosis signal cascade [23,24].

Currently, there are still no effective treatment options for TBI. Most interventions focus on rehabilitation therapies to improve cognitive function of the patients, but no effective therapies have been developed to counteract apoptosis in the affected brain areas. In this aspect, Notch inhibitor DAPT may benefit the therapy of TBI by inhibiting neuron apoptosis following TBI. In addition, a previous study reported that Hes1 knockdown promoted the differentiation of neural precursor cells into mature neurons and improved spatial learning and memory capacity of adult mice following TBI [25]. These findings are consistent with our results that DAPT downregulated Notch and Hes1 and improved neurological function and cognitive function following TBI.

## Conclusions

Notch signaling is activated in the acute phase of brain trauma and plays a detrimental role. The activation of Notch signaling can be used as an indicator of the severity of acute craniocerebral trauma. In addition, Notch inhibitor DAPT may be used as a potential drug for the treatment of acute traumatic brain injury.

## Supporting information

S1 Checklist The ARRIVE guidelines checklist. (PDF)
